# Diesel Exhaust Inhalation Elicits Acute Vasoconstriction *in Vivo*

**DOI:** 10.1289/ehp.11027

**Published:** 2008-03-18

**Authors:** Alon Peretz, Jeffrey H. Sullivan, Daniel F. Leotta, Carol A. Trenga, Fiona N. Sands, Jason Allen, Chris Carlsten, Charles W. Wilkinson, Edward A. Gill, Joel D. Kaufman

**Affiliations:** 1 Occupational and Environmental Medicine Program, Department of Environmental and Occupational Health Sciences; 2 Department of Surgery (Vascular Surgery); 3 Department of Medicine and; 4 Department of Psychiatry and Behavioral Sciences, University of Washington, Seattle, Washington, USA

**Keywords:** air pollution, brachial artery, catecholamines, endothelin-1, vasoconstriction

## Abstract

**Background:**

Traffic-related air pollution is consistently associated with cardiovascular morbidity and mortality. Recent human and animal studies suggest that exposure to air pollutants affects vascular function. Diesel exhaust (DE) is a major source of traffic-related air pollution.

**Objectives:**

Our goal was to study the effects of short-term exposure to DE on vascular reactivity and on mediators of vascular tone.

**Methods:**

In a double-blind, crossover, controlled exposure study, 27 adult volunteers (10 healthy and 17 with metabolic syndrome) were exposed in randomized order to filtered air (FA) and each of two levels of diluted DE (100 or 200 μg/m^3^ of fine particulate matter) in 2-hr sessions. Before and after each exposure, we assessed the brachial artery diameter (BAd) by B-mode ultrasound and collected blood samples for endothelin-1 (ET-1) and catecholamines. Postexposure we also assessed endothelium-dependent flow-mediated dilation (FMD).

**Results:**

Compared with FA, DE at 200 μg/m^3^ elicited a decrease in BAd (0.11 mm; 95% confidence interval, 0.02–0.18), and the effect appeared linearly dose related with a smaller effect at 100 μg/m^3^. Plasma levels of ET-1 increased after 200 μg/m^3^ DE but not after FA (*p* = 0.01). There was no consistent impact of DE on plasma catecholamines or FMD.

**Conclusions:**

These results demonstrate that short-term exposure to DE is associated with acute endothelial response and vasoconstriction of a conductance artery. Elucidation of the signaling pathways controlling vascular tone that underlie this observation requires further study.

The association between ambient fine particulate matter (PM) air pollution [particles with aerodynamic diameter ≤ 2.5 μm (PM_2.5_)] and increased cardiovascular morbidity and mortality in epidemiologic studies (Dominici et al. 2006; Miller et al. 2007; Pope et al. 2002) has led to investigation of underlying mechanisms, which remain cryptic (Bhatnagar 2006). Several studies link ambient (especially combustion-derived) PM with increased vascular tone (Brook et al. 2002; Campen et al. 2005; Proctor et al. 2006), and animal studies showed possible involvement of sympathetic nervous system activation (Proctor et al. 2006) and vasoconstriction pathways (Bouthillier et al. 1998; Li et al. 2005). Evidence suggests that the effect of PM on vascular reactivity may be greater in individuals at risk for coronary artery disease (O’Neill et al. 2005). Human studies to date have provided limited insight into the mechanism of the vascular effects of PM, although recent evidence has documented enhanced exercise-induced ischemia with controlled exposure to diesel exhaust (DE) (Mills et al. 2007).

DE is a complex mixture of particulate and gaseous pollutants that comprise a substantial and biologically active fraction of urban ambient air pollution (Health Effects Institute 1995) and is a very appropriate experimental model exposure, especially with the current interest in traffic-related air pollution. Evidence suggests that inhalation of DE is associated with perturbation in endothelial function (Campen et al. 2005; Mills et al. 2005; Tornqvist et al. 2007). We exposed young adults, healthy or with metabolic syndrome, to DE under controlled settings. Young adults with metabolic syndrome have increased subclinical atherosclerosis (Tzou et al. 2005) and are at increased risk for cardiovascular morbidity and mortality (Isomaa et al. 2001). We focused on individuals with metabolic syndrome because they might be more susceptible to DE effects. We hypothesized that DE inhalation would result in vaso-constriction and decreased endothelium-dependent flow-mediated dilation (FMD) of the brachial artery within 30 min of exposure. We also hypothesized that vascular effects of DE might be related to activation of the sympathetic nervous system or increased release of the endothelium-dependent vasoconstrictor endothelin-1 (ET-1).

## Materials and Methods

We conducted a crossover, double-blind experiment, randomized to order, of DE inhalation and a sham [filtered air (FA) only] exposure, with each participant exposed on three different days to each of three conditions: FA, and DE calibrated to 100 μg/m^3^ (DE_100_) and 200 μg/m3 (DE_200_) PM_2.5_.

### Study participants

Participants were recruited if they were 18–49 years of age, non-smokers for at least 6 months, with no history of ongoing medical care for heart disease, hypertension, asthma, diabetes, hypercholesterolemia, or other chronic condition; all had normal spirometry. We separately recruited and randomized participants who were healthy or had metabolic syndrome. Healthy participants had body mass index (BMI) < 30 kg/m^2^, fasting blood sugar < 126 mg/dL, no signs of arrhythmia or ischemia on electrocardiogram (ECG), and blood pressure < 130/85 mmHg. Metabolic syndrome participants fulfilled any three of the following five criteria: waist circumference ≥ 102 cm in males and ≥ 88 cm in females; triglycerides ≥ 150 mg/dL; HDL cholesterol < 40 mg/dL in males and < 50 mg/dL in females; systolic blood pressure ≥ 130 mmHg or diastolic blood pressure ≥ 85 mmHg; fasting glucose ≥ 100 mg/dL (Grundy et al. 2005).

All participants gave written informed consent. The consent form and study protocol were approved by the University of Washington Human Subjects Division.

### Exposure session protocol

Exposures began at approximately 0900 hours and were 2 hr in duration; participants were at rest throughout. Exposures were separated by at least 2 weeks. Women were exposed only during the first 2 weeks of the menstrual cycle, after the end of menstrual flow, to comprise the follicular phase. Participants fasted (other than water) for at least 10 hr before each visit and throughout the study session. On arrival, an intravenous catheter was placed in the left arm of the participant for preexposure and subsequent blood draws. About 30 min later, we measured pre-exposure brachial artery diameter (BAd) on the participant’s right arm. Thirty minutes after the exposure ended, BAd was measured again, along with response to inflation of a cuff on the proximal arm (FMD). A postexposure blood draw was performed at 3 hr from the onset of exposure (~ 30 min after ultrasound measurement). Arterial blood pressure was measured one time before each BAd measurement on the participant’s left arm.

### Exposure system

Characteristics of the exposure system have been described (Gould et al. 2008). Briefly, DE was derived from a 2002 model turbocharged direct-injection 5.9-L Cummins B-series engine (6BT5.9G6; Cummins, Inc., Columbus, IN) in a generator set. Load was maintained at 75% of rated capacity, using a load-adjusting load bank (Simplex, Springfield, IL) throughout the exposures. We used no. 2 undyed, on-highway fuel and Valvoline 15W-40 crankcase oil. Fuel deliveries were regularly tested for aromatics and sulfur content. Emissions were diluted in two phases, resulting in final breathing zone concentrations that could be calibrated between 15 and 400 μg/m^3^ PM_2.5_. Crankcase emissions were not entrained into the exposure system. All dilution air for the system was passed through HEPA and carbon filters, permitting an FA control exposure option with very low particulate and gaseous organic pollutant levels. The air entering the exposure room was conditioned to 18°C and 60% relative humidity. During exposures, PM_2.5_ concentrations were continuously measured and dynamically adjusted to maintain steady-state conditions [tapered element oscillating microbalance (TEOM); 1400a PM2.5; Rupprecht & Patashnick Co., Albany, NY]. Multistage samples collected on a micro-orifice uniform deposition impactor (MOUDI; MSP, Shoreview, MN) indicated a mass median diameter of 0.104 μm (geometric SD = 1.15) determined from 100 and 200 μg/m^3^ sample sessions. The facility maintains low concentrations of the gaseous copollutants carbon monoxide and nitrogen dioxide (Table 1). One-minute averages (± SE) of particle numbers (particles per cubic centimeter) during exposure (condensation particle counter, model 3022A; TSI, Inc., Shoreview, MN) were 2889.5 ± 780.3 for FA; 30590.2 ± 2427.6 for DE_100_; and 52840.9 ± 6860.9 for DE_200_.

### Brachial artery studies

All measurements of BAd and FMD were performed using previously described methods (Corretti et al. 2002; Peretz et al. 2007). Studies were performed in a quiet and dark room and at controlled ambient temperatures between 20 and 26°C. Blood pressure was measured on the left arm before each study. The electrocardiogram was continuously monitored. In supine position, and after 10 min of rest, the right arm of the participant was comfortably immobilized in the extended position, allowing for ultrasound scanning of the brachial artery above the antecubital fossa. The location of the ultrasound probe during the participant’s first test was recorded and used for later tests in that subject.

One experienced sonographer, blinded to exposure concentrations, scanned the brachial artery in longitudinal orientation using an HDI 5000 Ultrasound Instrument (Philips Medical Systems, Bothell, WA) with a 5–12 MHz linear array transducer. In each examination, preexposure recording of vessel images followed baseline Doppler assessment of blood flow velocity. After exposure, vessel images were again recorded, followed by inflation of a cuff on the upper arm to 50 mmHg above systolic pressure for 5 min. The blood pressure cuff was placed proximal to the location of the ultrasound probe. Blood flow velocity was then obtained after cuff release, and the brachial artery was imaged and recorded for 5 min. Images were digitized from the video output of the ultrasound machine using a frame grabber under control of custom software on a personal computer. Image acquisition was gated with ECG signal to capture end diastole in each cardiac cycle.

BAd was assessed by a single trained operator using a validated and automated, beat-by-beat image-processing software package (Vascular Tools 4.6; Medical Imaging Applications, Coralville, IA) previously introduced (Sonka and Lauer 2002) and independently validated (Mancini et al. 2002; Preik et al. 2000). Briefly, the operator defined a vascular region of interest, which was then applied automatically to identify the media-to-media diameters in each frame over time. We excluded from analysis those frames with detected vessel border < 70% of the width of the region of interest (confidence index < 70%) and, for postcuff deflation sessions only, frames with diameters that differed by > 1 SD from a polynomial fit. Custom software developed in the MATLAB programming environment (MathWorks, Natick, MA) was used to extract measurements from the time series of diameter measurements. We previously reported a small day-to-day variability of baseline BAd for the described methods (coefficient of variation = 5.1 ± 1.7%) (Peretz et al. 2007).

We determined changes in BAd after each exposure session by calculating the difference between postexposure measurement and pre-exposure measurement. Decreases in BAd after exposures were considered vasoconstriction, whereas increases were termed vasodilation. FMD was calculated as percent difference of the maximal diameter after cuff deflation from the postexposure BAd.

### Plasma ET-1

Whole blood samples were collected in potassium citrate; plasma was aliquoted and frozen at –70°C within 30 min of blood draw, and batch run. After extraction from plasma, ET-1 levels were measured by ELISA (R&D systems, Minneapolis, MN). We assayed only samples collected at FA and DE_200_ exposure sessions. We report a mean concentration of ET-1 in picograms per milliliter.

### Plasma catecholamines and metabolites

To assess activity of the sympathetic nervous system at serial time points, we assayed plasma levels of catecholamine precursor l-3-4-dihydroxyphenylalanine (l-DOPA); catecholamines norepinephrine (NE), epinephrine, and dopamine; deaminated metabolites of NE-dihydroxyphenylglycol (DHPG); and dopamine dihydroxyphenylacetic acid (DOPAC). Whole blood samples were collected in lithium heparin and chilled immediately after draw. Within 15 min of sample collection, plasma was separated (1,000 × *g*, 2,400 rpm, CR412 centrifuge, 4°C, 10 min) and stored in sterile polypropylene cryotubes at –80°C. Plasma was extracted with adaptation of alumina method of Anton and Sayre (1962) (ESA, Inc., Chelmsford, MA), separated by high-performance liquid chromatography using a reverse-phase C-18 column, and measured by electrochemical detection (Coulechem II; ESA, Inc.). The coefficients of variation (CV) for the assays used are 7.05% [intraassay CV, based on intraassay variation in internal reference standard 3,4-dihydroxy-benzylamine (DHBA)] and 10.81% (interassay CV, based on pooled plasma samples repeated in each assay). We assayed only samples collected at FA and DE_200_ exposure sessions.

### Statistical analysis

All statistical testing was based on two-tailed *p*-values with α = 0.05. Descriptive data are presented as mean ± SE unless specified otherwise. Statistical analyses were performed using STATA 9.1 (StataCorp., College Station, TX).

Plasma catecholamines values were natural log-transformed for analysis. Changes attributable to exposure are the difference between postexposure and preexposure for BAd, ET-1, and catecholamines. Comparisons of study vascular and plasma end points between DE and FA exposures were made by paired *t*-test. We used analysis of variance (ANOVA) to compare averages of preexposure BAd between exposures. We tested for effect modification by subject-related characteristics and for period and carryover effects, using ANOVA.

## Results

Twenty-seven participants completed all three exposure sessions: 10 healthy and 17 with metabolic syndrome, with characteristics shown in Table 2.

### Vessel caliber studies

Data were not available from four healthy participants for pre-exposure BAd; one participant had missing preexposure BAd data from the FA exposure session; and one participant had missing pre-exposure and postexposure BAd data from the DE_100_ exposure session.

Overall, 22 participants had paired pre-exposure and postexposure BAd data for the FA and DE_200_ exposure comparisons, and 21 participants had data for the FA and DE_100_ exposure comparisons. The average preexposure BAd was not statistically different between FA (3.94 mm), DE_100_ (4.06 mm), and DE_200_ (4.09 mm) (*p* = 0.6).

**Figure 1:**
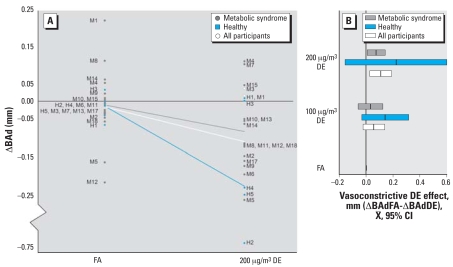
Changes in BAd after exposures. CI, confidence interval. (*A*) Individual changes in BAd after exposures to 200 μg/m^3^ DE or FA. The difference in millimeters in resting BAd between postexposure and preexposure (ΔBAd) in each of the two exposure conditions is shown. Individual subjects are designated by M for metabolic syndrome or H for healthy. The lines demonstrate mean ΔBAd at each exposure level. (*B*) Dose–response relationship of DE effect on BAd. Bars for each DE exposure concentration demonstrate the mean and 95% CI of DE vasoconstrictive effect in reference to FA represented as no vasoconstriction (X ^–^ = 0) for the two study subpopulations and the overall group. Wide CIs for the healthy group reflect the small sample size and not higher variance.

Average BAd measures before and after exposures are presented in Table 3. Of the 22 participants with paired BAd measurements in both DE_200_ and FA, 16 had smaller vessel caliber after DE than FA: 4 demonstrated vasoconstriction with DE and dilation with FA, 10 demonstrated greater degree of vasoconstriction with DE than FA, and 2 demonstrated blunted vasodilation with DE compared with FA (Figure 1A). Overall, exposure to DE_200_ was associated with an average 0.11-mm greater degree of constriction of the brachial artery than exposure to FA (*p* = 0.01). Exposure to DE_100_ was also associated with a greater degree of arterial constriction than exposure to FA (–0.05 mm), but the difference was not statistically significant. Of note, the point estimate of this effect is half that observed with DE_200_, making a linear dose–response effect the most likely explanation (Figure 1B). Age, sex, BMI, fasting plasma lipid levels, carryover, and period effects did not modify the association between DE and vasoconstriction. There was no effect of DE on systolic or diastolic blood pressure (data not shown).

### FMD

All 27 participants had paired FMD data for FA and DE_200_ exposure comparisons, and 26 participants had data for FA and DE_100_ exposure comparisons (Table 3). Exposure to DE_200_ elicited a greater, but not statistically significant, FMD (16.0%) compared with FA (13.9%) (*p* = 0.07). A similar effect was observed comparing DE_100_ and FA. Health status of participants did not affect the differences in FMD between DE and FA.

### Plasma analytes

**Figure 2:**
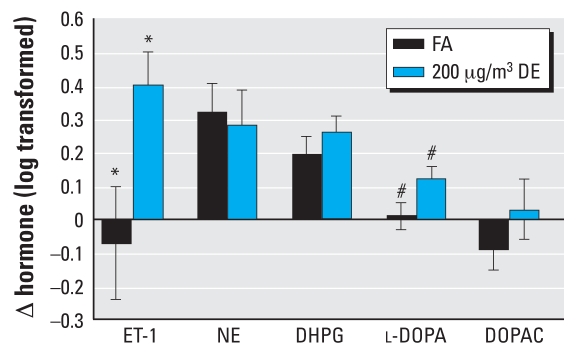
Changes in hormonal levels from pre-exposure. Mean changes (95% CIs) of log-transformed hormonal levels from preexposure to 3 hr from initiating exposure to 200 μg/m^3^ DE or FA. *p = 0.01. ^#^*p* < 0.05.

Averages of plasma hormones for exposures to DE_200_ and FA are shown in Table 4. Twenty-two participants (6 healthy and 16 metabolic syndrome) had paired preexposure and postexposure ET-1 samples from both DE_200_ and FA exposures. Exposure to DE_200_ was associated with an increase in plasma ET-1 levels at 3 hr [Δ = 0.43; 95% confidence interval (CI), 0.25 to 0.61], whereas the levels were nearly unchanged after FA (Δ = 0.01; 95% CI, –0.28 to 0.31) (Figure 2). The impact of DE_200_ on plasma ET-1 compared with FA was statistically significant (*p* = 0.01). Healthy participants had, on average, greater increments in plasma levels of ET-1 after DE_200_ (Δ = 0.76; 95% CI, 0.38 to 1.1) compared with participants with metabolic syndrome (Δ = 0.31; 95% CI, 0.12 to 0.51) (*p* = 0.02).

We compared ET-1 response between vasoconstrictors (participants who demonstrated vasoconstriction with exposure to DE_200_ or more vasoconstriction from DE_200_ than from FA) (*n* = 14) and nonvasoconstrictors (*n* = 8). The increment in plasma ET-1 3 hr after exposure to DE_200_ was greater in vasoconstrictors than nonvasoconstrictors, but not significant. Plasma levels of ET-1 at 3 hr were not different between DE_200_ (0.33 pg/mL) and FA (0.34 pg/mL) for nonvasoconstrictors (*p* = 0.9), whereas vasoconstrictors had higher levels 3 hr post-DE_200_ (0.38 pg/mL) compared with FA (0.26 pg/mL) (*p* = 0.06).

Because of insufficient sample availability, only 13 participants (5 healthy and 8 metabolic syndrome) had paired preexposure and post-exposure sympathetic marker samples from both DE_200_ and FA exposures. Two subjects with extremely high values of DHPG (for one) and DOPAC (for the other) were considered outliers and were excluded from the statistical analysis. Epinephrine and dopamine were not compared between FA and DE because of a very low signal-to-noise ratio of the assay.

Except for NE, all sympathetic plasma markers tended to be increased at 3 hr after exposure to DE_200_ to a greater extent than the changes after exposure to FA (Figure 2). Only the difference in exposure-related changes of l-DOPA (Δ = 0.11; 95% CI, 0.01 to 0.22) was statistically significant.

## Discussion

In this study we demonstrated that controlled exposure to DE, a major source of urban air pollution, acutely affects vascular tone in humans. These results add to previous evidence of vascular dysfunction after exposure to DE (Mills et al. 2005) and vasoconstriction associated with pollutant particles in humans (Brook et al. 2002) and animal models (Batalha et al. 2002; Huang et al. 2002; Proctor et al. 2006). Our data suggest, for the first time in humans, that endothelins including ET-1 may be involved in the vascular effect of DE. This provides a potentially vital insight into the pathways responsible for cardiovascular health effects of combustion-derived air pollution.

Although the absolute magnitude of DE-induced brachial artery vasoconstriction was small (ranging from 1.2 to 13.9% of luminal caliber), the effect was notably consistent, suggested a dose–response relationship, and was comparable in magnitude to abnormal coronary vasoconstriction induced by increasing doses of acetylcholine (Anderson et al. 1995; Hambrecht et al. 2000; Takase et al. 1998). In addition, similar magnitude of vasoconstriction was found in healthy young adults exposed to concentrated ambient fine particles and ozone (Brook et al. 2002). Although we do not postulate that DE-induced vasoconstriction of this magnitude induces acute cardiac events, this striking phenomenon and other sequelae sharing signaling pathways could result in plaque destabilization, vasospasm, and proarrhythmogenic processes to explain the consistent epidemiologic observations of air pollution-induced acute cardiovascular effects. Further, a range of reactions likely occurs in the large populations exposed to ambient air pollution, so that a larger magnitude of vasoconstriction—including clinically significant vasoconstriction in the most susceptible individuals—could be anticipated. Finally, small changes seen in epicardial coronaries or brachial vessels, although not causative of myocardial infarction, reflect processes that are also occurring in the microvasculature, leading to arteriolar spasm and capillary derecruitment, with resulting myocardial ischemia. The recent findings by Mills et al. (2007) demonstrating exercise-induced myocardial ischemia with a similar exposure (DE at 300 μg/m^3^) underscore the relevance of this finding.

Although individuals with metabolic syndrome are at higher risk for cardiovascular morbidity, and *a priori* were presumed by us to be more susceptible to the vascular effects of DE, we observed greater vasoconstriction after exposure to DE in the healthy participants. This may be artifactual because of the small number of healthy participants with available preexposure and postexposure BAd and the higher variability of baseline BAd compared with participants with metabolic syndrome (Table 3). However, there may be biologic reasons for this effect. Healthy individuals may have more vessel plasticity, exhibiting a greater range of normal vascular response. Metabolic syndrome is associated with chronic arterial stiffness and a tonic constriction of the coronary arteries (Setty et al. 2003), and thus may be less responsive to a transient insult derived from a short-term exposure to DE. Metabolic syndrome is characterized by chronic oxidative stress (Van Guilder et al. 2006), possibly with consequent up-regulation of antioxidant mechanisms; therefore, these individuals may be more protected from an oxidative insult induced by air pollutants. Finally, the effect of DE on plasma ET-1 was greater in healthy individuals, which may explain a greater vasoconstriction in those individuals.

We demonstrated that DE induces a measurable increase in ET-1 levels in humans, reflecting a perturbation in homeostatic control of vascular tone, which may have resulted in the observed brachial artery vasoconstriction. Previous studies have also demonstrated that the endothelin system may be affected after inhalation of cigarette smoke (Haak et al. 1994) and air pollutants. Investigations in rats (Bouthillier et al. 1998; Vincent et al. 2001) demonstrated an association between urban air pollution inhalation and increased circulating levels of ET-1, between diesel soot (but not carbon black) and plasma ET-3 (Vincent et al. 2001), and between on-road particles and ET-2 (Elder et al. 2004). Epidemiologic studies in children have corroborated the association between air pollution and circulating ET-1 (Calderon-Garciduenas et al. 2003). Vincent et al. (2001) showed that increased levels of plasma ET-1 were associated with increased systemic blood pressure, suggesting a role of endothelins in modulating the vascular effects. In an *ex vivo* exposure to soluble components of DE, coronary arteries from ApoE^–/–^ mice showed an enhanced response to ET-1 (Campen et al. 2005).

Increased plasma concentrations of ET-1 can result from either increased synthesis or decreased clearance. Although we did not assess ET-1 precursor, which would have provided a more direct evidence of increased ET-1 synthesis, there is little reason to speculate that decreased clearance occurs as a result of DE exposure. Increased *de novo* synthesis of ET-1 can occur as result of increased expression of the preproET-1 in the endothelium of any vascular bed, and increased circulating ET-1 from DE could be attributed easily to either pulmonary vasculature or systemic arterial endothelium. Our observations do not provide insight into whether increased ET-1 was attributable to direct pollutant effect on systemic vascular endothelium, increased ET-1 generated in the pulmonary vasculature, or a downstream effect due to other circulating mediators elaborated because of effects in the lung. Thompson et al. (2004) showed increments in both plasma ET-1 and preproET-1 mRNA levels in the lungs of rats exposed to urban particles. To support the hypothesis that DE may induce pulmonary synthesis of ET-1 are the facts that endothelium-derived ET-1 is primarily secreted into the abluminal space (Wagner et al. 1992), and most of it is cleared from the plasma very fast (Vierhapper et al. 1990).

It is possible that increased ET-1 synthesis from DE represents a secondary phenomenon, such as from stimulation by catecholamines (Levin 1995). It was suggested that ambient pollutant particles effect vascular tone through augmentation of sympathetic activity (Huang and Ghio 2006), such as by activation of α1 receptors on vascular smooth muscle cells. Although plasma catecholamine measures are problematic (Boomsma et al. 1993; Goldstein et al. 2003), potential involvement of the autonomic nervous system in the response to DE cannot be excluded by our data.

Observation of a higher FMD in a conduit artery after DE compared with FA does not support the thesis associating DE particles primarily with inhibition of nitric oxide synthase in endothelium (Ikeda 1998; Rajagopalan et al. 2005). This does not exclude nitric oxide–mediated effect of DE on resistance vasculature as demonstrated by Mills et al. (2005). Moreover, our study does not exclude the possibility that DE causes decreased NO bioavailability in association with the observed increase in ET-1. We cannot exclude non-endothelium-dependent mechanisms mediating the greater hyperemic vasodilation of the brachial artery after DE. Furthermore, it is possible that even in the setting of DE-induced depletion of NO, other DE-related factors may have contributed to a later rise in NO compared with FA. Under this assumption, *a*) the higher ET-1 levels after exposure to DE may have acted on endothelial ET_B_ receptors, causing direct stimulation of nitric oxide synthase activity (Cardillo et al. 2000); *b*) the initiation of vasoconstriction served as stimulus on the endothelium to release NO (Harris and Matthews 2004); and *c*) possible altered expression of α2B-adreno-ceptors may have influenced nitric oxide synthesis (Duling et al. 2006).

### Study limitations

Several limitations in the study require consideration. First, only a small number of healthy participants had available preexposure and postexposure BAd data. This fact, and the relatively high variability of BAd in those participants, may have influenced the greater apparent effect of DE on BAd in those participants compared with participants with metabolic syndrome. It should not be concluded that healthy subjects are more susceptible.

Furthermore, our observations do not confirm an increase in ET-1 synthesis, because the precursor molecule was not measured. In addition, because of limitations related to experiments involving human participants, ET-1 was measured from the systemic circulation, where its concentrations are expected to be different from those at the level of the target tissue in the arterial circulation.

Finally, in the absence of an endothelium-independent vasodilator, we cannot determine conclusively that the greater maximal vasodilatation after exposure to DE was not endothelium dependent.

## Conclusions

This study demonstrates a DE effect on control of conduit arterial tone involving endothelins in human subjects and suggests a safe experimental model for further research on cardiovascular effects of traffic-related air pollution. Taken together with other research, this experimental approach, with rigorous control over exposure situations and potentially confounding factors, supports the importance of this observation in elucidating a significant mechanistic pathway underlying the consistent epidemiologic evidence of air pollution-induced cardiovascular morbidity and mortality.

## Figures and Tables

**Table 1:** Average PM_2.5_ mass concentrations^a^ and gas concentrations^b^ measured during 2-hr exposure sessions of 27 participants.

Pollutant	FA	100 μg/m^3^ DE	200 μg/m^3^ DE
PM_2.5_ (μg/m^3^)	4.60	101.53	205.33
NO_2_ (ppb)	15.89	16.53	24.67
NO (ppb)	40.49	958.14	1537.93
CO (ppm)	0.27	0.51	0.89

a From TEOM; discrete 10-min averaging intervals.

b 1-min averaging intervals.

**Table 2:** Characteristics of study participants (*n* = 27) by health status.

Characteristic	Healthy	Metabolic syndrome
Participants (no.)	10	17
Age, years [mean (range)]	29.8 (20–42)	38.5 (20–48)
Sex [female:male (no.)]	2:8	6:11
Race (no.)		
Caucasian	7	13
African American	2	2
Asian	1	1
Other	0	1
Body mass index (kg/m^2^)	24.5 ± 0.5*	42 ± 1.9*
Blood pressure (mmHg)^a^		
Systolic	108 ± 3.8*	123 ± 2.2*
Diastolic	75 ± 2.9*	83 ± 1.6*
Total cholesterol (mg/dL)	151.1 ± 9.9*	194.8 ± 7.7*
Triglycerides (mg/dL)	70.8 ± 13.6*	177 ± 27.1*
Glucose (mg/dL)	88.9 ± 1.9*	99.7 ± 2.6*

Where not specified, values are mean ± SE.

a Measure at preexposure.

* *p* < 0.01.

**Table 3:** BAd at baseline and after flow-mediated dilation (mean ± SE).

	Healthy	Metabolic syndrome	All
BAd (mm)	*n* = 5	*n* = 16/17^a^	*n* = 21/22^a^
FA			
Preexposure	3.48 ± 0.26	4.08 ± 0.14	3.94 ± 0.14
Postexposure	3.46 ± 0.25	4.07 ± 0.15	3.93 ± 0.14
100 μg/m^3^ DE			
Preexposure	3.79 ± 0.31	4.15 ± 0.15	4.06 ± 0.13
Postexposure	3.64 ± 0.29	4.10 ± 0.16	3.99 ± 0.14
200 μg/m^3^ DE			
Preexposure	3.88 ± 0.39	4.15 ± 0.14	4.09 ± 0.14
Postexposure	3.64 ± 0.29	4.07 ± 0.15	3.97 ± 0.13
FMD (%)	*n* = 10	*n* = 16/17^a^	*n* = 26/27^a^
FA	15.0 ± 1.7	13.3 ± 1.1	13.9 ± 0.9
100 μg/m^3^ DE	15.8 ± 1.3	16.2 ± 1.3	16.1 ± 1.1
200 μg/m^3^ DE	16.9 ± 2.0	15.5 ± 1.1	16.0 ± 1.0

a Sixteen participants with metabolic syndrome had available paired data for DE at 100 μg/m^3^ and FA comparisons; 17 had available paired data for DE at 200 μg/m^3^ and FA. The total number of subjects with paired data is smaller for DE at 100 μg/m^3^ and FA comparisons versus DE at 200 μg/m^3^ and FA comparisons.

**Table 4:** Levels of plasma ET-1 and catecholamines (pg/mL).^a^

	FA		200 μg/m^3^ DE	
Biomarker	Preexposure	Postexposure	Preexposure	Postexposure
ET-1				
Healthy (*n* = 6)	1.47 ± 0.21	1.35 ± 0.27	0.86 ± 0.06	1.62 ± 0.18
Metabolic syndrome (*n* = 16)	1.35 ± 0.17	1.41 ± 0.15	1.31 ± 0.18	1.62 ± 0.18
All participants (*n* = 22)	1.38 ± 0.13	1.39 ± 0.13	1.19 ± 0.14	1.62 ± 0.13
NE				
Healthy (*n* = 5)	128.6 (0.8)	217.4 (1.1)	122.0 (0.9)	227.1 (0.9)
Metabolic syndrome *n* = 8)	182.7 (0.7)	225.8 (0.7)	175.6 (0.7)	191.8 (0.6)
All participants (*n* = 13)	161.2 (0.6)	222.7 (0.7)	154.2 (0.6)	203.7 (0.6)
DHPG				
Healthy (*n* = 4)	1090. (0.6)	1294.6 (0.9)	1071.1 (0.7)	1475.6 (0.8)
Metabolic syndrome (*n* = 8)	919.3 (0.6)	1120.6 (0.7)	830.6 (0.6)	1055.4 (0.6)
All participants (*n* = 12)	968.7 (0.6)	1171.5 (0.6)	898.2 (0.6)	1170.1 (0.6)
l-DOPA				
Healthy (*n* = 5)	1483.4 (0.7)	1435.5 (0.8)	1474.1 (0.7)	1681.6 (0.7)
Metabolic syndrome (*n* = 8)	1544.2 (0.6)	1604.7 (0.6)	1363.8 (0.6)	1532.1 (0.6)
All participants (*n* = 13)	1522.2 (0.6)	1542.1 (0.6)	1402.2 (0.6)	1583.9 (0.6)
DOPAC				
Healthy (*n* = 5)	2386.2 (0.7)	2011.6 (0.7)	2878.4 (0.8)	2313.2 (0.9)
Metabolic syndrome (*n* = 7)	1469.7 (0.6)	1412.0 (0.6)	1614.2 (0.9)	1945.1 (0.9)
All participants (*n* = 12)	1770.8 (0.6)	1671.9 (0.6)	2013.4 (0.7)	2079.2 (0.7)

a Catecholamines (all biomarkers except endothelin-1, which is mean ± SE) are presented as geometric mean and geometric standard error of the mean, which is a factor multiplication.
